# G-Quadruplexes Abet Neuronal Burnout in ALS and FTD

**DOI:** 10.3390/antiox15010005

**Published:** 2025-12-19

**Authors:** Alan Herbert

**Affiliations:** Discovery, InsideOutBio, 42 8th Street, Unit 3412, Charlestown, MA 02129, USA; alan.herbert@insideoutbio.com

**Keywords:** flipons, ALS, TDP43, C9ORF72, G-quadruplex, heme, oxidative stress, FTD, Amyloid-beta, dementia

## Abstract

Expansion of d(GGGGC)_n_ repeat in the C9ORF72 gene is causal for Amyotrophic Lateral Sclerosis (ALS) and Frontal Temporal Dementia (FTD). Proposed mechanisms include Repeat-Associated Non-AUG translation or the formation of G-quadruplexes (GQ) that disrupt translation, induce protein aggregation, sequester RNA processing factors, or alter RNA editing. Here, I show, using AlphaFold V3 (AF3) modeling, that the TAR DNA-binding protein (TDP-43) docks to a complex of GQ and hemin. TDP-43 methionines lie over hemin and likely squelch the generation of superoxide by the porphyrin-bound Fe. These TDP-43 methionines are frequently altered in ALS patients. Tau protein, a variant of which causes ALS, also binds to GQ and heme and positions methionines to detoxify peroxides. Full-length Tau, which is often considered prone to aggregation and a prion-like disease agent, can bind to an array composed of multiple GQs as a fully folded protein. In ALS and FTD, loss-of-function variants cause an uncompensated surplus of superoxide, which sparks neuronal cell death. In Alzheimer’s Disease (AD) patients, GQ and heme complexes bound by β-amyloid 42 (Aβ4) are also likely to generate superoxides. Collectively, these neuropathologies have proven difficult to treat. The current synthesis provides a framework for designing future therapeutics.

## 1. Introduction

Here, I initially focus on the poorly understood interaction between TAR DNA-binding protein (*TARDBP*, TDP-43) variants and the *C9ORF72* d(GGGGC)_n_ repeat expansion. Variants of both are associated with an increased risk of Amyotrophic Lateral Sclerosis (ALS) [1,2]. I will describe how folding the *C9ORF72* repeat sequence RNA into GQ enables the binding of heme groups, which, in turn, generate superoxides. I propose that the docking of wild-type TDP-43 squelches this process. Furthermore, TDP-43 variants that no longer suppress peroxide production increase the risk of ALS, leading to neuronal oxidative damage similar to that seen with the loss-of-function (LOF) superoxide dismutase (*SOD1*) variants that are causal for ALS. The modelling reveals that the Tau protein (encoded by MAPT) can also interact with hemin and GQ to suppress superoxide production. LOF *TAU* variants necessary to squelch peroxidase production are causal for Frontotemporal Dementia (FTD). Similarly, GQ and hemin complexes stabilized by Aβ4 and transition metals also appear likely to generate superoxides and cause neuronal damage. The findings exemplify how repeat sequences, called flipons, affect disease susceptibility by the structures they form.

## 2. ALS Associated with *C9ORF72*

ALS is a highly debilitating disease. The average survival is 3–5 years following the onset of motor neuron degeneration. The lifetime risk of the disease approaches 1:400 [3,4]. About 10% of Amyotrophic Lateral Sclerosis (ALS) cases occur in families harboring causal variants, including due to an expansion of the d(GGGGC)_n_ repeat present in *C9ORF72*. The usual number of d(GGGGC)_n_ repeats in the population is around 2, while 30 or more are commonly found in patients [5,6]. The expanded repeats have attracted attention because they can form alternative DNA structures, including hairpin loops, three-stranded triplexes (TPX), and four-stranded DNA quadruplexes (dGQs), as well as RNA G-quadruplexes (rGQs), which are the focus here [7,8,9]. Such alternative folds are encoded by sequences called flipons that enable the dynamic regulation of cellular responses [10].

When flipon repeats expand, they can freeze in an alternative conformation, disrupting transcription and replication of the host gene by impeding the processivity of RNA and DNA polymerases. DNA damage and truncated transcripts then accrue. With *C9ORF72*, the expanded repeat segment undergoes bidirectional transcription. Translation of these RNA fragments produces Repeat-Associated Non-ATG (RAN) peptides that are neurotoxic in model organisms [11,12]. The transcripts can also sponge up various factors involved in RNA processing, and scaffold nuclear and cytoplasmic aggregates. Complete loss-of-function (LOF) in *C9ORF2*-knockout mouse models produces a fatal autoimmune disease [13]. These outcomes have recently been reviewed [14].

## 3. ALS, FTD, and TDP-43

One protein that plays a prominent role in these neurological diseases is TDP-43. Aggregates of TDP-43 characterize frontotemporal dementia (FTD), Alzheimer’s disease, and limbic predominant age-related TDP-43 encephalopathy (LATE) [15]. Each disease has a characteristic distribution of aggregates: in FTD, they are found in the neurons of the cortex and hippocampus, while in ALS, they form in spinal cord motor neurons. The formation of aggregates containing both TDP-43 and r(GGGGC)_n_ repeat RNAs reinforces the interrelated roles these polymers play in the pathogenesis of ALS, with oxidative stress further accelerating disease onset [15,16].

**Figure 1:**
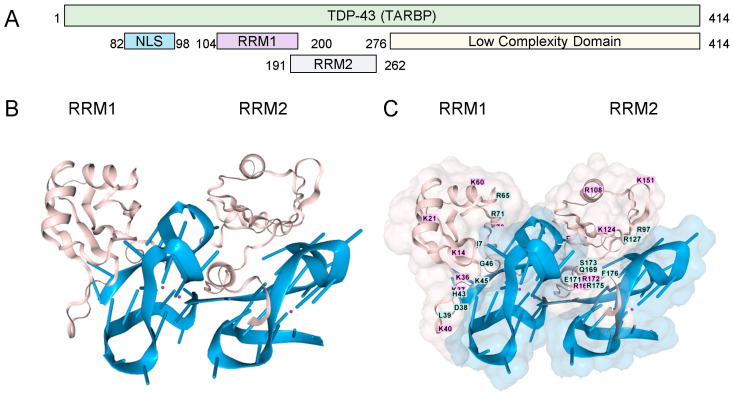
TDP-43 and RNA G-Quadruplexes. (**A**). The TDP-43 domain structure (**B**). Cartoon representation of the interaction of the two RRMs ( pink coloring, residues 101-280, UNIPROT Q13148, accessed 17 August 2025), each bound to a parallel-stranded rGQ (each colored blue). The amino (**C**). A space-filling representation of the interaction with labeling of the amino acids in the RRM domains. (RRM: RNA Recognition Motif; NLS: Nuclear Localization Sequence; GQ Sequence (A(AGGGUU)_4_AGGGUUAGGGUUAGGGU)_2_).

TDP-43 has two RNA Recognition Motifs (RRMs) (residues 101–280) and a low-complexity carboxy-terminal region prone to aggregation (Figure 1A). The binding of the RRMs to rGQ has been experimentally demonstrated [17]. We modeled this interaction using AF3, which shows the binding of each RRM domain to a separate rGQ, both of which are formed in a single RNA (Figure 1B). The TDP-43 residues are mapped in Figure 1C. Each rGQ is composed of a stack of three guanosine RNA tetrads, each consisting of four bases that hydrogen-bond to one another through both their Hoogsteen faces. The rGQ sequence corresponds to the human telomere repeat sequence. The four RNA strands run parallel to each other, yielding a rGQ structure that preferentially binds TDP-43 [18], and one that AF3 correctly modeled.

These interactions with rGQ are consistent with models in which TDP-43 binds and masks rGQs, preventing the sequestration of other rGQ-binding proteins involved in splicing and RNA editing by the double-stranded RNA deaminases [19]. LOF TDP-43 variants would then alter numerous pathways and disrupt the Q/R editing essential for restricting calcium permeability of the GluR-B receptor (encoded by GRIA2) [20].

## 4. rGQ and the Low Complexity Domain of TDP-43

**Figure 2:**
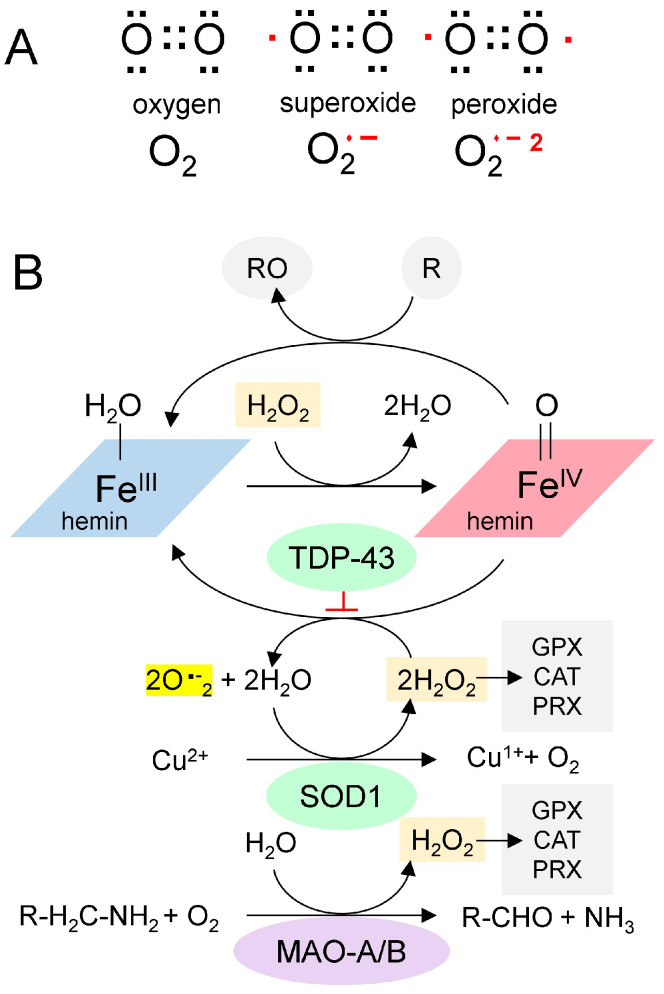
The Chemistry of Reactive Oxygen Species. (**A**). Paired (black dots) and unpaired electrons (red dots) in reactive oxygen species. (**B**). A proposed scheme for the generation of superoxide from GQ-bound hemin (adapted from [30]). Hemin bound to GQ catalyzes the production of superoxide and the oxidation of proteins (indicated by “R”). SOD1 is a copper-based catalyst that dismutates superoxide into hydrogen peroxide and oxygen. Monoamine oxidases consume hydrogen peroxide during the deamination of monoamine transmitters in neurons. It is proposed here that TDP-43 traps the superoxides generated by the hemin-GQ complex. (CAT: catalase; GPX: Glutathione peroxidase; PRX: Peroxiredoxin).

In addition to proteins, rGQs bind small planar compounds that dock onto their end caps, including hemin, which is present at high concentrations within cells. The interaction of hemin with rGQs is of particular interest because the complex acts as a peroxidase, generating reactive oxygen species that are damaging to cells (Figure 2A). This reactivity has been documented both in vitro and within cells [21,22]. The reduced form of hemin is called heme, an essential component of the mitochondrial electron transport chain complexes II, III, and IV. These proteins couple redox reactions that fully reduce molecular oxygen to water, with disruptions to the chain resulting in superoxide generation. Notably, recent findings show that superoxide leakage from complex III in rats with neuronal complex IV deficiency recapitulates ALS [23]. Under normal circumstances, these superoxides are removed by superoxide dismutase enzymes, such as SOD1 (Figure 2B). However, human LOF variants of *SOD1* are causative for ALS. Overall, these findings demonstrate a central role for oxidative stress in ALS disease progression. 

Heme and hemin cellular concentrations are influenced by many factors [24]. Heme binds to membranes via hydrophobic interactions and to proteins via anionic groups. Transporters regulate hemin uptake into cells and lysosomes, as well as its export [25]. Proteins such as heme binding protein 1 (HEBP1) also bind hemin and translocate it to lysosomes, where proteolysis generates an immunoactive N-terminus fragment [26]. Cytoplasmic hemin levels regulate heme synthesis by controlling the turnover of BTB domain and CNC homolog 1 (BACH1) by inducing ubiquitylation and proteolysis of BACH1 [27]. Otherwise, BACH1 binds DNA antioxidant response element motifs, preventing nuclear factor erythroid 2-related factor 2 (NRF2) from inducting heme oxygenase-1 (HO-1) production, an enzyme that breaks down heme. HO-1 is located mainly in the endoplasmic reticulum, with the iron released by degradation of hemin sequestered into storage proteins, primarily ferritin [28]. Collectively, these pathways help regulate intracellular heme levels and the cell’s redox state. In diseases where heme accumulates, such as porphyria, the breakdown products of the tetrazole ring can also contribute to the disease presentation [29].

Redox (oxidation-reduction) pathways also moderate the effects of hemin. Enzymes such as catalase, glutathione peroxidase, and peroxiredoxin remove hydrogen peroxide and protect cells. In neurons, a potent source of hydrogen peroxide is monoamine oxidase (MAO), which deaminates neurotransmitters (Figure 2B). Potentially, chronic oxidative stress from MAO or other sources can lead to recurrent mitochondrial damage, thereby further increasing hemin release and creating a positive feedback cycle. Eventually, the cycle overwhelms the antioxidant defenses, resulting in rapid cell death.

**Figure 3:**
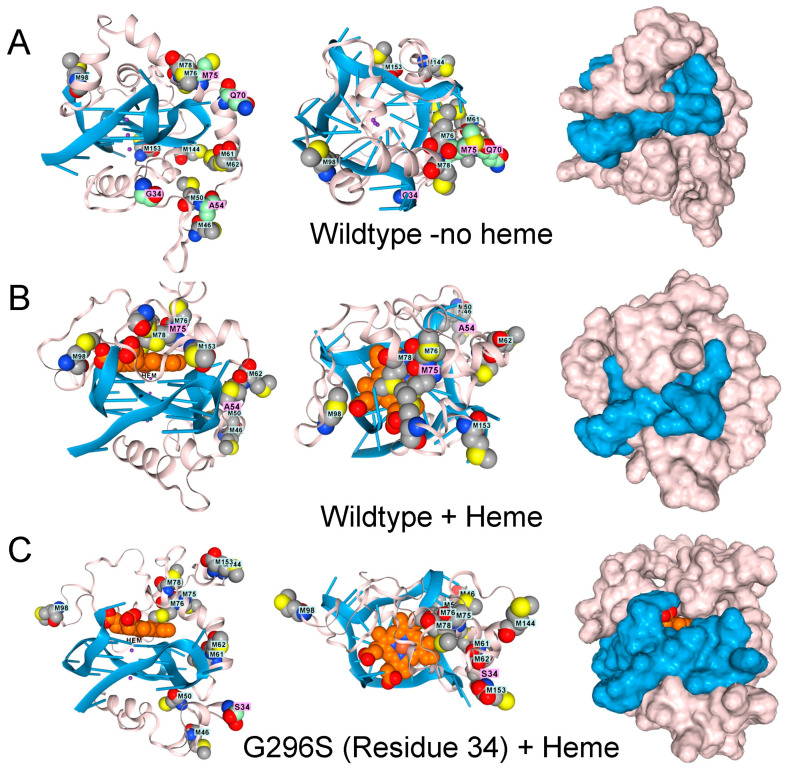

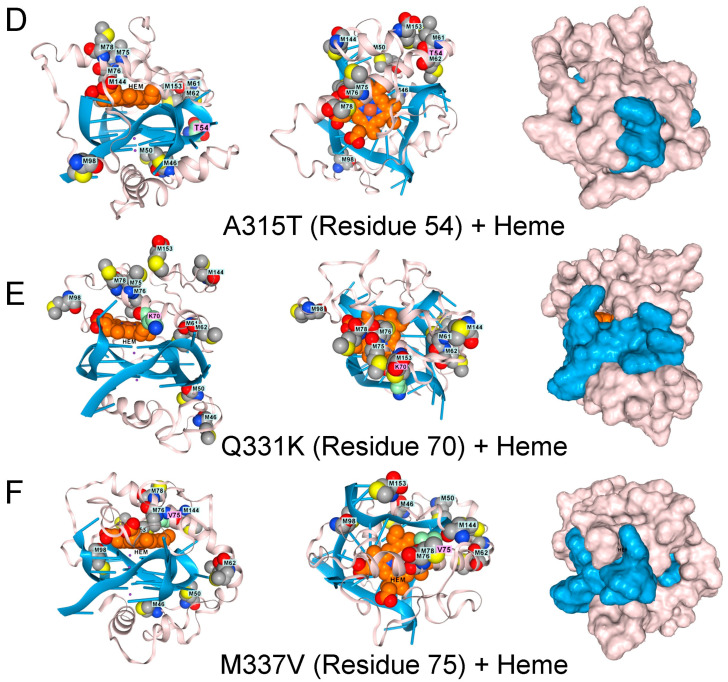
The low complexity region of TDP-43 binds to GQ. (**A**). Docking of the wildtype sequence to a parallel strand GQ in the absence of hemin. Methionine residues are shown in space fill. The green-shaded residues vary in disease, as shown in panels (**C**–**F**). Side, top-down, and surface images are shown. (**B**). The complex also binds a heme group, shown in light brown and situated atop the GQ stack, which is caged by methionines (space-filling model) that can capture free radicals. Mutations to TDP-43 that are linked to ALS disrupt the methionine cage. (**C**). G295S (residue 34 in the model). (**D**). A315T (residue 54 in the model). (**E**). Q331K (residue 70 in the model) (**F**). M336V (residue 75 in the model).

Interestingly, in AF3, the low-complexity domain (LCD) of TDP-43 (amino acids 262–414) docks to rGQ and fully folds rather than forming an array of parallel β-sheets, as described in the literature (Figure 3A) [31]. The docking of TDP-43 to rGQ is enhanced by heme (Figure 3B), consistent with an earlier experimental analysis [32]. Modeling of the protein, heme, rGQ complex reveals that the TDP-43 methionines are positioned to squelch the reaction (Figure 3B), a role that methionine is exquisitely suited to play [33]. These oxidized methionines can then be recovered by a family of methionine sulfoxide reductases (MSR) that restore the native protein [34]. 

TDP-43 variants that disrupt the positioning of the methionines are found in ALS patients, as shown by AF3 renders for the G295S. A315T, Q300K, and M336V substitutions (Figure 3D–F). The genetic findings support a model in which methionines in the LCD of TDP-43 protect cells by minimizing damage caused by superoxide radicals generated by a rGQ-hemin peroxidase. The C9ORF72 repeat expansion produces transcripts that favor the formation of these GQ-hemin peroxidase arrays in the cytoplasm. The arrays will also oxidize hemin, promoting its turnover. The iron released will be captured by ferritin, which likely explains the elevated levels observed in patients [35]. Intracellular hemin biomarkers provide a way to measure this form of oxidative stress and may be a useful tool for measuring disease progression in ALS patients [25].

**Figure 4:**
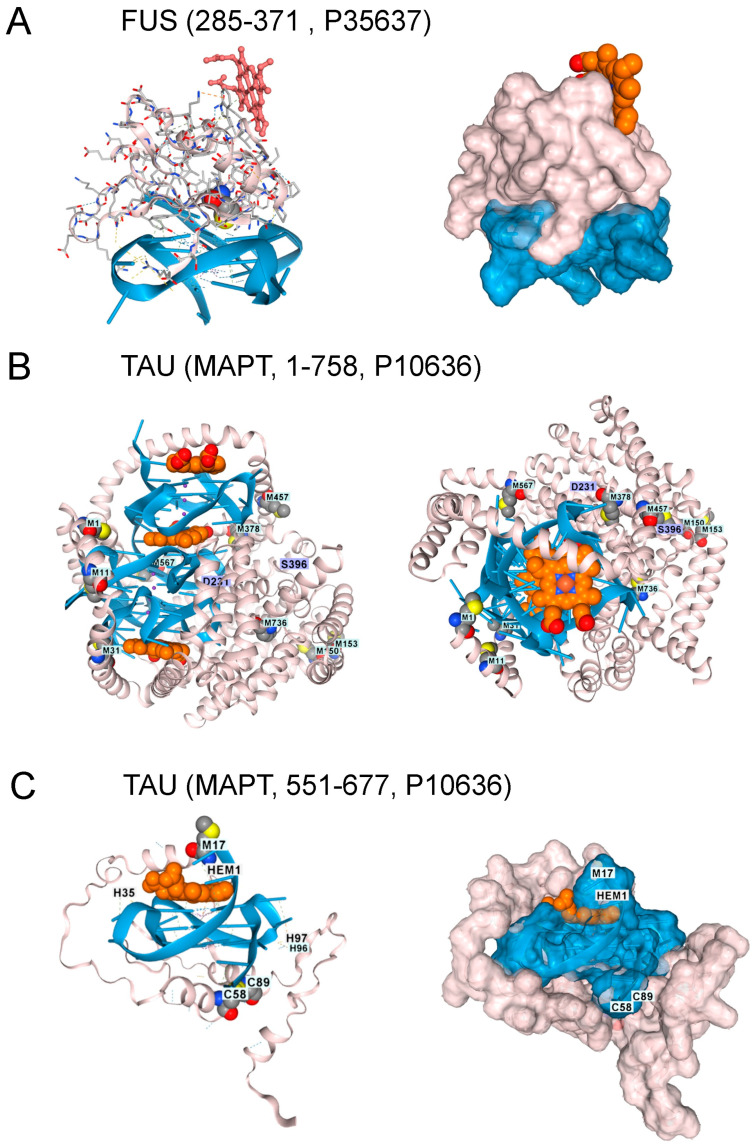
Other Proteins Altering the Risk of ALS and FTD. 1. (**A**). The FUS protein RNA-binding domain (residues 285–371, UNIPROT P35637) also docked to GQ, but the interaction excludes hemin. Surface model for the interaction shown in panel A. The GQ is in blue, the protein in blush, and hemin in burnt orange. (**B**). With AF3 Tau, the protein (residues 1–758, UNIPROT P10636) only folded in the presence of both GQ and heme. Here, docking of Tau (blush) to an RNA composed of 3 telomeric repeats (blue), with the location of three heme (burnt orange) docking sites and the positions of methionines in the protein shown. A top view of the complex. Phosphorylation of residues D231 and S396 is associated with GQ-containing fibrils in disease patients. (**C**). Docking of a single hemin to Tau residues 551-677 bound to a single telomeric GQ. C58 and C89 (corresponding to C608 and C639 in the intact protein) form a disulfide bond. M17 (corresponding to M567) sits over the heme group as it does in the intact protein. The surface representation of the TAU complex is shown in the right panel (UNIPROT, last accessed 17 August 2025).

Translocated in liposarcoma protein FUS protein variants are also linked to ALS and FTD [36,37]. AF3 models show that FUS also binds to the GQ produced by the C9ORF72 flipon repeat [38]. However, docking FUS onto rGQ does not accommodate heme within the fold (Figure 4A). FUS engagement by rGQ is then likely to protect against hemin-catalyzed superoxide production. A deficiency of FUS protein may increase the risk of ALS and FTD by removing a restriction to rGQ-hemin peroxidase formation.

## 5. rGQ, TAU, and Aβ4

Tau protein aggregates are a feature of FTD and are associated with hyperphosphorylated proteins, an outcome that hemin has been reported to promote [39]. Indeed, hemin is essential for Tau docking to telomeric rGQs in AF3, with no Tau folding observed when either hemin or rGQ is omitted from the model. The fold can accommodate multiple rGQ repeats, with a hemin stacked between each quadruplex. A complex of Tau with three hemin and three rGQ repeats is shown in Figure 4B. Interestingly, the binding to a single telomeric repeat to Tau is mediated by the microtubule binding domain (MTBD) (residues 551–677, UNIPROT P10636). The location of methionine in the intact protein and of M567 in the MTBD does offer some protection against heme-generated superoxide. Notably, an increase in Tau phosphorylation at S396 and T231 (Figure 4C) is associated with higher rGQ formation in Alzheimer’s Disease [40], potentially due to their stabilization of the rGQ binding fold through ionic interactions. A disulfide bond formed between C608 and C639 is also likely to stabilize the binding of the MTBD to a rGQ under oxidative conditions (Figure 4C).

**Figure 5:**
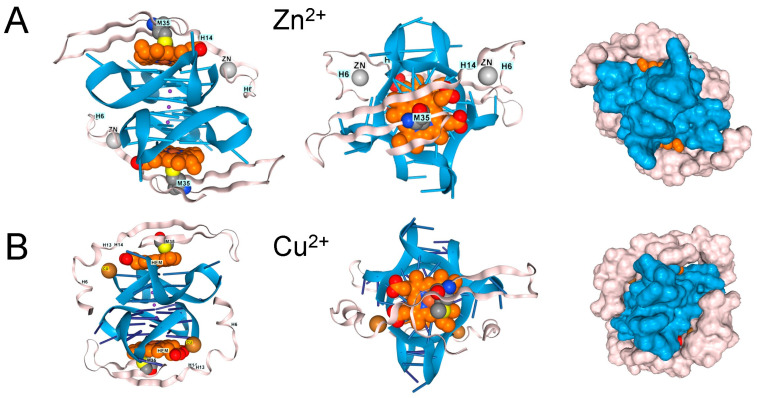

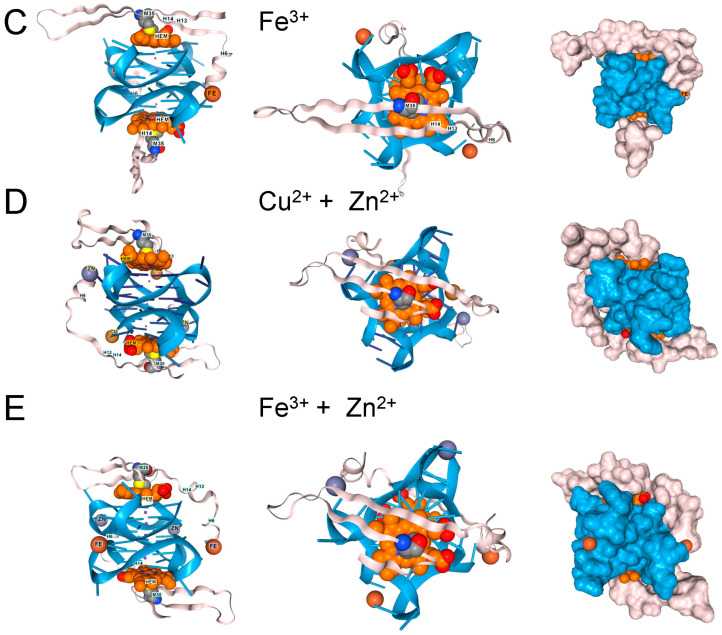
β-Amyloid 42 fragments (residues 672–713 of Amyloid-beta precursor protein (UniProt P05067) associate with GQ and heme in the presence of (**A**). Zn^2+^, (**B**). Cu^2+,^ and (**C**). Fe^3+^, but the peptide only contains a single methionine. The binding of one metal does not preclude the binding of another. (**D**). Binding of copper and zinc ions. (**E**). Binding of ferric and Zinc ions. The proximity of a binding site to DNA, along with an ion’s potential to generate reactive oxygen species, may influence its effect on disease processes. In the cartoons on the left, hemin is shown in orange, the GQ in blue, and the protein chain in pink. The images on the right are space-fill models of the complexes.

In contrast, the single methionine in Aβ4 (M35) appears insufficient to inhibit the peroxidase activity of hemin bound to rGQ. Instead, the complex can dock additional transition metal ions with redox functionality that can also oxidize Y10 to generate dityrosine cross-links (Figure 5) [41]. Both the M35 oxidation and the association of AD with reduced MSR activity have been well-documented over the years, as has the association of Aβ4 with hemin, and the peroxidase activity of the complex [41,42,43,44,45].

## 6. Targeting Superoxide Production

The proposed mechanism relies on the chemistry of rGQs. It exemplifies how flipon state alters disease susceptibility—the formation of stable GQ arrays that bind hemin and produce large amounts of superoxide. Cells are damaged in the same manner as with SOD1 LOF variants, ultimately leading to a positive feedback loop that results in the catastrophic loss of metabolically active neurons. Patients would benefit from therapies that prevent GQ-bound hemin from producing superoxide. Small-molecule ligands that displace hemin from quadruplexes have been experimentally shown to inhibit superoxide production [22]. However, those compounds that act by binding to rGQ will likely interfere with the usual roles of rGQ and dGQ in a cell.

Other compounds that inhibit rGQ formation by the C9ORF72 repeat, by stabilizing the hairpin fold, offer an alternative strategy, as they may also prevent RAN translation [46]. Overexpression of FUS to diminish the binding of TDP-43 and Tau to rGQ risks disrupting other cellular processes [47]. Partial knockdown of C9ORF72 disrupts its normal function in cells and impairs autophagy [48]. Drugs targeting Fe in hemin risk disrupting the catalytic copper of SOD1 (Figure 2B). Diminishing Fe^IV^-catalyzed superoxide formation may also inhibit its removal by Cu^2+^ reduction, with the effects counteracting each other. Notably, the use of the metal chelator deferiprone accelerated the decline in Alzheimer’s Disease [49]. In addition to Fe, the drug also has a high affinity for Cu [50]. MAO inhibitors that prevent H_2_O_2_ generation offer another approach to reducing superoxide production; however, they have not been proven to slow ALS progression [51]. Recent strategies in ALS treatment have focused on developing multifunctional drugs that combine FeIII-dependent oxidative stress inhibitors [48] and MAO inhibitors [49] into a single compound [52,53]. In general, caution may be exercised with such drugs, given the challenges in using them for the routine treatment of depression in cases of neurodegeneration [54]. Effective dosing may also depend on the development of biomarkers to track their efficacy in reducing superoxide production while maintaining SOD1 activity. Other treatment approaches could target HEBP1 to minimize production of the immunoactive N-terminal peptide that could amplify oxidative stress by activating microglia [26]. In the 3 × Tg-Alzheimer’s Disease model, *HEBP1* knockout in hippocampal neurons by CRISPR/Cas9 reduced neuronal cell death, perhaps by preventing export of this peptide from lysosomes, where it is produced [55]. Increased clearance of the GQ-hemin complexes released by dying cells would also help improve outcomes by preventing the spread of oxidative stress to nearby cells.

## 7. Conclusions

The critical role of oxidative stress in degenerative disease has been known for many years [56]. Genetic studies have revealed how protein-coding mutations can lead to gain-of-function mutations that generate free radicals, while others affect antioxidant pathways [57]. The findings here reveal how flipons affect disease susceptibility by the structures they form. In this model of ALS and FTD, a noncoding RNA composed of the C9ORF72 repeat sequence folds into a G-quadruplex that sequesters hemin, forming a peroxidase that generates oxidative stress. The onset of disease occurs when cells can no longer compensate. The defenses that protect the cell depend in part on methionine-rich antioxidant proteins such as TDP-43 and TAU, variants of which increase the risk of ALS and FTD. Flipons are challenging targets due to their deep embedding within a cell’s biology. However, RNA-based strategies, such as RNAi and RNA-guided gene editing, offer a new therapeutic approach. These interventions are designed to prevent the folding of C9ORF72 transcripts into G-quadruplexes that bind hemin to form extended peroxidase arrays. By doing so, they protect against the subset of flipon diseases that cause neurological disease by generating overwhelming oxidative stress [58,59,60].

## 8. Methods

Models were generated using the AlphaFold V3 (AF3) web interface (https://alphafoldserver.com/, last accessed 1 December 2025). The exact conditions and seed sequences used are given in the titles of the PDB files in the Supplementary Data File. AF3is not explicitly trained to model flipon structures, nor their interactions with protein motifs. However, the model can be nudged to explore these structures by varying the input conditions and specifying the seed used to build the model. AF3 was also run to test the effect of residue mutation on the docking interactions. AF3 generates five different models for each run. The model selected was based on the bonding scheme and steric fit. Interestingly, proteins with intrinsically disordered regions and known not to fold in AF3 properly did so in the presence of the rGQ ligand. Previous studies have shown that these models selected using these criteria are stable in molecular dynamics simulations [19]. Figures were prepared using the web implementation of NGL Viewer (https://nglviewer.org/ngl/, last accessed 30 October 2025) [61].

## Data Availability

PDB files for the structures presented in the figures are supplied as a Supplemental Data File.

## Supplementary Materials

The following supporting information is available at: https://www.mdpi.com/article/10.3390/antiox15010005/s1, Herbert_GQ_ALS_FTD.zip. The archive contains PDB files for the structures presented in the figures.
